# Downregulation of metastasis suppressor 1(MTSS1) is associated with nodal metastasis and poor outcome in Chinese patients with gastric cancer

**DOI:** 10.1186/1471-2407-10-428

**Published:** 2010-08-15

**Authors:** Ke Liu, Gefang Wang, Houzhong Ding, Ying Chen, Guanzhen Yu, Jiejun Wang

**Affiliations:** 1 Department of Medical Oncology, Changzheng Hospital, Shanghai, China; 2 Department of Oncology, the Eighty-fifth Hospital, Shanghai, China; 3 Department of Surgery, the first People's Hospital, Kunshan, Jiangsu, China; 4 Department of Pathology, Changhai Hospital, Shanghai, China

## Abstract

**Background:**

The putative tumor metastasis suppressor 1(MTSS1) is an actin-binding scaffold protein that has been implicated to play an important role in carcinogenesis and cancer metastasis, yet its role in the development of gastric cancer has not been well illustrated. In this study, we detected MTSS1 expression and explored its clinical significance in gastric cancer.

**Methods:**

Immunohistochemistry was performed using tissue microarrays containing gastric adenocarcinoma specimens from 1,072 Chinese patients with normal adjacent mucosa, primary gastric cancer and lymph node (LN) metastasis and specific antibody against MTSS1. MTSS1 mRNA and protein expression were detected by reverse transcription-polymerase chain reaction and Western blotting. The clinical follow-up was done in the 669 patients living in Shanghai that was chose from the 1072 cases.

**Results:**

Complete loss of MTSS1 expression was observed in 751 cases (70.1%) of the 1,072 primary tumors and 103 (88%) of 117 nodal metastases; and loss of MTSS1 expression was significantly associated with poorly differentiated tumors, large tumor size, deep invasion level, the presence of nodal metastases and advanced disease stage. Moreover, multivariate analysis demonstrated that loss of MTSS1 expression correlated significantly with poor survival rates (RR = 0.194, 95% CI = 0.144-0.261, P < 0.001).

**Conclusions:**

MTSS1 expression decreased significantly as gastric cancer progressed and metastasized, suggesting MTSS1 may serve as a useful biomarker for the prediction of outcome of gastric cancer.

## Background

Gastric carcinoma (GC), which is the second most common cause of cancer-related death in the world, deprives more than 700,000 lives per annum [1]. Its incidence varies considerably worldwide and has recently been decreasing in developed countries, but remains stably in developing countries [2-4]. Furthermore, the fact that gastric cancer is insensitive to conventional chemotherapy and is rarely amenable to radiotherapy leaves the survival durations in patients with gastric cancer unchanged in recent years. This highlights the need for the determination of prognostic factors predicting the outcome and the development of novel therapeutic strategies. Previous studies have indicated that disease stage and lymph node metastasis are the most important prognostic factors in gastric cancer. Moreover, some molecular markers have been identified and attempted to use clinically [5-7]. Nevertheless, other potential prognostic factors related to survival in these patients remain unclear.

Metastasis suppressor 1 (MTSS1), also known as MIM (missing in metastasis), was originally identified by *Lee et al *[8] as a potential metastasis suppressor gene that was present in non-metastatic bladder cancer cell lines, but was not expressed in a metastatic bladder cancer cell line. This gene, mapped to human chromosome 8q24.1, encodes a 5.3 kb mRNA and a polypeptide predicted to be an actin-binding protein of 356 amino acids with homology to the WASp (Wiscott-Aldrich Syndrome protein) family [8]. Functional analyses of MTSS1 have shown that MTSS1 induced actin-rich protrusions resembling microspikes and lamellipodia at the plasma membrane and promoted disassembly of actin stress fibres [9]. Actin filament assembly is associated with cytoskeletal structure organization and many forms of cell motility [10]. These data have suggested that MTSS1 protein may be important in regulating cytoskeletal dynamics, and as a consequence it would play a potential role in the invasion and metastatic behaviour of cancer cells.

The study surrounding MTSS1 is quite small, yet this protein has been the subject of controversy. Preliminary analysis by Northern blotting demonstrated that MTSS1 is widely expressed but is most abundant in spleen, thymus, testis, and prostate, with low levels also detected in uterus and colon [8]. Since this pioneering article, other reports have indicated that MTSS1 played a role as a metastasis suppressor in prostate cancer [11,12], bladder cancer [8,11,13] and benign lesions, but up-regulated in basal cell carcinomas [14]. However, other evidences showed that MTSS1 is unlikely to be a metastasis suppressor. It acts as a scaffold protein that interacts with actin-associated proteins to modulate lamellipodia formation [15]. *Ma et al *suggests that MTSS1 is a regulator of carcinogenesis in hepatocellular carcinoma [16]. And it is a member of the sonic hedgehog (SHH) signalling pathway that modulates Gli responses during growth and carcinogenesis [14].

Although these studies cited above suggested MTSS1 as a promising candidate biomarker and playing an important role in tumorigenesis, little is known about the function of MTSS1 in gastric cancer. In our study, we sought to determine the expression of MTSS1 in resected gastric cancers and investigate the correlation of MTSS1 expression and clinicopathologic features and survival, in an attempt to discover the potential influence of MTSS1 on the development of gastric cancer.

## Methods

### Patient specimens

A total of 1,072 patients with gastric cancer who underwent curative surgery at Changhai Hospital in Shanghai, People's Republic of China, from 2001 to 2005 were enrolled in this study. Mean age of these patients was 59 years old; 757(71%) were male and 315 (29%) were female. All the other clinicopathological characteristics of these patients could be found in previous study [17]. Clinical follow-up results were available for the 669 patients from the Shanghai area (mean follow-up duration, 40 months [range, 1-110 months]). Total number of death event was 380 cases, and 289 cases are still alive. All of the tissue specimens were obtained for the present study with patient informed consent, and the use of the human specimens was approved by the Changhai Hospital Institutional Review Board.

### Immunohistochemistry and evaluation of immunostaining

Tissue sections were deparaffinized in xylene, and then rehydrated in graded concentrations of ethyl alcohol (100%, 95%, 75%, then water). TMA sections were microwave-treated twice in citrate buffer (PH 6.0) at 99°C for 6 min. The sections were placed in 3% H_2_O_2 _for 10 min to inhibit the endogenous peroxide activity, washed three times with phosphate-buffered saline (PBS) buffer for 5 min and placed in normal goat serum as blocking antibody at room temperature for 10 min. The primary antibodies used were ab56780 (Abnova, Caltag-Medsystems Ltd., Buckingham, UK, 1:50) for MTSS1. After incubation at 4°C for 24 h, sections were washed three times with PBS buffer for 10 min. Biotinylated anti-mouse/rabbit immunoglobulin was used as the second antibody. 3, 3-Diaminobenzidine (DAB) was used as a chromogen. The sections were counter-stained with hematoxylin.

All slices were evaluated without knowledge of the clinical outcome. MTSS1 protein expression in the 1,072 cases was evaluated by two individuals (G. Y. and Y. C.) under an Olympus CX31 microscope (Olympus, Center Valley, PA). Sections were considered positive for MTSS1 when more than 5% of tumor cells were stained in the cell cytoplasm. Staining was scored independently by the two individuals who were blinded to each other's findings.

### RNA preparation and reverse transcription- polymerase chain reaction

Total cellular RNA was isolated from the homogenised gastric samples using the AB gene Total RNA Isolation Reagent (Advanced Biotechnologies Ltd., Epsom, Surrey, UK). RNA concentration and quality were determined through spectrophotometric measurement (WPA UV 1101, Biotech Photometer, Cambridge, UK). cDNA was generated from 1 ug of each RNA sample and a reverse transcribed using a transcription kit (Takara, Kyoto, Japan). The quality of DNA was verified using β-actin primers (sense:GCTGTCACCTTCACCGTTC; antisense:CCATCGTCCACCGCAAAT). MTSS1 mRNA levels were assessed using MTSS1 primers: (sense: TGG GTCCACTGAGCCCCACACATTGTTG and antisense: GGTGGCCATTGTGGG GTGGAATG -AA). PCR amplification was carried out with Ex Taq DNA polymerase reaction system (Takara, Kyoto, Japan). Conditions for PCR were 94°C 4 min,30 s at 94°C for denaturation, 30 s at 60°C for annealing and 90 s at 72°C for elongation (30 cycles). PCR products were electrophoresed through 1.5% agarose gels and analyzed by computerized densitometric scanning of the images using the Quantity-One imaging software normalized with internal β-actin control.

### Western Blotting Assay

Whole-cell lysates were prepared from human gastric cancer and normal gastric tissue specimens. Standard Western blotting was performed using a mouse monoclonal antibody against human MTSS1 in a 1:50 dilution (Abnova, Caltag-Medsystems Ltd., Buckingham, UK) and an anti-mouse/rabbit immunoglobulin (ZB-2305, Jackson, America). Equal protein sample loading was monitored by probing the same membrane filter with an anti-β-actin antibody. The probe proteins were detected using the Amersham enhanced chemiluminescence system according to the manufacturer's instructions.

### Statistical analysis

Association among factors was evaluated by the Pearson χ2 test. Within-group correlations of continuous and ordinal variables were assessed using Pearson's correlation coefficient or Spearman's rank correlation coefficient when appropriate. Differences between samples mRNA and Western Blotting were assessed by paired t-test. Survival curves were calculated according to the Kaplan-Meier method. Survival data shown in this study were pertaining to overall survival. To this end, non-gastric cancer deaths were considered as lost to follow up as of time of death in the statistical analysis. Differences between survival curves were examined with the log-rank test. Multivariate analysis of prognostic factors related to overall survival was carried out using Cox's proportional hazards model and a stepwise procedure. The covariates included sex, age, histological classification, Lauren's classification, tumor size, location of tumor, depth of invasion, lymph node metastasis, and disease stage. The accepted level of significance was P < 0.05. Statistical analyses and graphics were performed with the SPSS 13.0 statistical package (SPSS, Inc., Chicago, IL).

## Results

### MTSS1 expression in patients with gastric cancer

**Figure 1:**
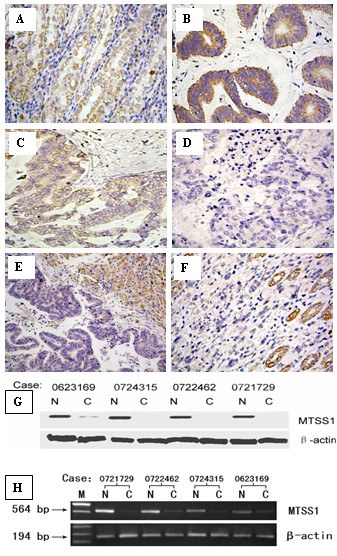
**Analysis of MTSS1 expression in human gastric cancers and adjacent normal mucosa specimens**. A: Normal (nonneoplastic) gastric mucosa; B and C: Gastric cancer positive expression of MTSS1 in well-(B) and moderate-(C) differentiated tumors. D: Gastric cancer negative MTSS1 expression; E: MTSS1 expression in the hepatic metastasis and adjacent nonneoplastic liver tissues; F: MTSS1 expression in gastric cancer and adjacent nonneoplastic mucosa tissues; G and H: Western blotting analysis (G) and RT-PCR analysis (H) of MTSS1 in GC tissues and surrounding nontumor tissues, β-actin as a parallel control, N: surrounding nontumor tissues; T: tumor tissues.

All tissue microarray block sections selected for this study contained both normal and malignant epithelium. Figure 1 shows examples of tissue immunostained for the protein evaluated. MTSS1 immunostaining was of cytoplasm localization. The epithelium in normal mucosa specimens showed visible MTSS1 staining (Figure 1A). 751 cases (70.1%) showed negative staining of MTSS1 and the other 321 cases showed positive staining. These results were in concordance with that in the Western blotting study (Figure 1G). Importantly, we detected high expression of MTSS1 in adjacent normal epithelium but drastically reduced MTSS1 expression in the tumor cells (Figure 1F). Moreover, we observed that expression of MTSS1 varied with the different histological types. From the well and moderate histological levels to the histological undifferentiated, the expression of MTSS1 was gradually decreased (Figure 1B-D). In addition, MTSS1 expression was down-regulated in the liver metastases compared with the adjacent normal liver tissues, which serves as a positive control (Figure 1E).

Reverse transcription RT-PCR was used to evaluate MTSS1 expression level in 30 normal adjacent mucosa and 30 paired primary gastric cancer tissues. The result verified that MTSS1 expression level in GC tissues was significantly different from the paired normal adjacent mucosa. Consistent with the microarray data, this analysis showed that the MTSS1 expression mRNA level in GC was significantly lower than in normal adjacent mucosa. (P < 0.001) (Figure 1H).

### Correlation between MTSS1 expression and clinicopathologic characteristics of gastric cancer

The GC patients were divided into two groups according to the MTSS1 expression levels:

MTSS1 low or negative expressers (n = 751) and MTSS1 positive expressers (n = 321). Correlation between MTSS1 expression level and clinicopathologic characteristics of GC is summarized in Table 1. A statistically significant association was observed between MTSS1 expression level and tumor size, histology and Lauren's classification. In 634 cases well-differentiated to moderately differentiated tumors, 235 (37.1%) of the cases had positive MTSS1 expression in GC tissue; whereas in 438 poorly differentiated tumors, only 86 (19.6%) presented positive MTSS1 expression (P < 0.001). And in 618 cases of intestinal type in Lauren's classification, 211 (34.1%) presented positive MTSS1 expression but 110 (24.2%) cases with the diffuse-type showed positive. In the 825 cases of GC with tumor size (<6 cm), 265 (32.1%) presented MTSS1 expression, while only 56 (22.7%) of 247 cases of GC with tumor size (≥6 cm) presented MTSS1 expression (P = 0.005).

**Table 1:** Summary of clinicopathological parameters of gastric cancer and their correlation with loss of MTSS1 protein expression

Features	No. of patients (%)	Loss of MTSS1 expression (%)	P value
Tumor size			0.005
<6 cm	825(77.0%)	560(67.9%)	
≥6 cm	247(23.0%)	191(77.3%)	
Site			NS
Cardia and fundus	180(16.8%)	127(70.6%)	
Corpus	329(30.7%)	229(69.6%)	
Antrum	519(48.4%)	359(69.2%)	
Whole	44(4.1%)	36(81.8%)	
pT stage			< 0.001
T0-T2	312(29.1%)	173(55.4%)	
T3/T4	760(70.9%)	578(76.1%)	
pN stage			< 0.001
N0	356(33.2%)	205(57.6%)	
N1-3	716(66.8%)	546(76.3%)	
Disease stage			< 0.001
I/II	427(39.8%)	243(57%)	
III/IV	645(60.2%)	508(78.8%)	
Differentiation			< 0.001
Well/Moderate	634(59.1%)	399(62.9%)	
Poorly	438(40.9%)	352(80.4%)	
Lauren's classification			< 0.001
Intestinal-type	618(57.6%)	407(65.9%)	
Diffuse-type	454(42.4%)	344(75.8%)	
Total	1072	751(70.1%)	

We also observed that loss of MTSS1 expression correlated with advanced T, N, and TNM stage. Loss of MTSS1 expression occurred more frequently in large gastric tumors (invasion to level T3-T4) (76.1%) than in small ones (level T0-T2) (55.4%; P < 0.001) and more frequently in gastric tumors with regional LN metastasis (76.3%) than in N0-stage tumors (57.6%; P < 0.001). With regard to TNM stage, loss of MTSS1 expression was significantly associated with advanced disease stage: 78.8% at stage III -IV and 57% at stage I- II (P < 0.001).

### Relationship of MTSS1 protein expression in primary gastric tumors and LN metastases

In the tissue microarrays, 117 cases had available matched LN metastases. The MTSS1 expression rate in the metastases (12%) was lower than that in the primary tumors (26.5%) (P = 0.005).

### Relationship of loss of MTSS1 expression with poor outcome in patients with gastric cancer

**Figure 2:**
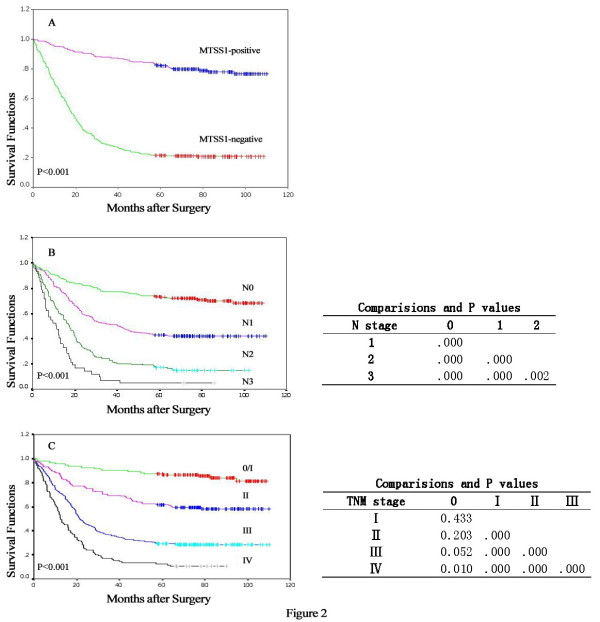
**Kaplan-Meier curves of survival durations in patients with gastric cancer treated with primary gastrectomy**. (A) Patients with cancers negative for MTSS1 expression had shorter survival durations than did patients with MTSS1 expression in their gastric cancers (P < 0.001). (B) The survival durations were significantly worse in patients with higher N stages than in those with lower N stages (P < 0.001). (C) The survival durations were significantly worse in patients with advanced stages than in those with early stages (P < 0.001).

**Figure 3:**
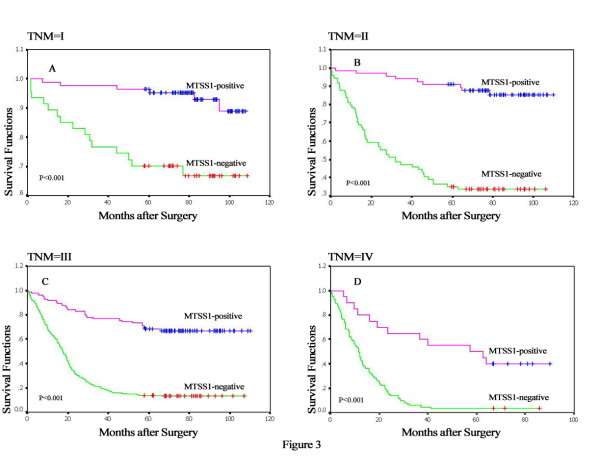
**Subgroup analysis of MTSS1 according to TNM**. (A-D) Stage-specific survival curves showed patients with loss of MTSS1 expression had poor survival to those with positive MTSS1 expression in each stage (P < 0.001).

Figure 2 presents several survival curves of the 669 GC patients, with the median cumulative survival duration in patients with resected gastric carcinoma of 40 months. Kaplan-Meier survival analyses revealed that the GC patients with loss MTSS1 expression had a significantly poor prognosis compared to those with positive MTSS1 expression (18 months versus 76 months; P < 0.001, Figure 2A). Furthermore, subgroup analysis of MTSS1 according to TNM was performed (Figure 3A-D). The outcomes of patients with MTSS1-negative expression were worse in each stage than that with MTSS1-positive expression (> 82 months for MTSS1-positive tumors *vs *72 months for MTSS1-negative tumors in stage I, P < 0.001; >78 months *vs *32 months in stage II, P < 0.001; >70 months *vs *18 months in stage III, P < 0.001; >57 months *vs *11 months in stage IV, P < 0.001). Aside from MTSS1 expression, survival analysis of other clinicopathological factors also revealed that lymph node metastases and clinical TNM stage were associated with prognosis of the patients with gastric cancer (Figure 2B.C).

Multivariate analysis using the Cox proportional hazards model for all of the significant variables in the univariate analysis showed that age, differentiation, nodal invasion, TNM stage and MTSS1 expression were independent prognostic factors (Table 2).

**Table 2:** Cox proportional hazards model analysis of prognostic factors

	B	SE	Wald	RR	95% CI		P value
Age	0.416	0.109	14.652	1.516	1.225	1.876	<.001
Tumor size	0.107	0.119	0.800	1.112	0.881	1.405	0.371
Differentiation	0.202	0.091	4.883	1.224	1.023	1.464	0.027
Location	0.028	0.068	0.172	1.029	0.900	1.176	0.678
Nodal metastasis	0.288	0.093	9.588	1.334	1.112	1.601	0.002
Gastric wall invasion	0.267	0.144	3.409	1.305	0.984	1.732	0.065
TNM	0.341	0.144	5.643	1.406	1.061	1.863	0.018
MTSS1	-1.640	0.152	116.494	0.194	0.144	0.261	<.001

SE, standard error

## Discussion

Although metastasis suppressor 1 (MTSS1) may be a critical regulator of carcinogenesis in different cancers, study of MTSS1 has been mainly restricted to several cancers and available data seem to be controversial, leaving the involvement of MTSS1 in cancer not clearly defined. Given that gastric cancer is one of the most malignant cancers in the world and that tumor recurrence and metastases are the major causes of death in patients with gastric cancer, resulting in a poor prognosis of the disease, we set out to investigate the role of MTSS1 in gastric cancer. In this study, we found that loss of MTSS1 expression was significantly associated with important clinical determinants of prognosis for gastric cancer including poorly differentiated tumors, diffuse-type in Lauren's classification, large tumor size, deep invasion level, the presence of nodal metastases and advanced disease stage. And patients without MTSS1 expression had shorter median survival durations than that with MTSS1 expression. Moreover, our immunohistochemistry data clearly revealed that MTSS1 expression significantly decreased from normal tissues to primary tumors to nodal metastases. To the best of our knowledge, this is the first clinical evidence that MTSS1 might play an important role in gastric cancer progression and metastasis.

Accumulating evidences suggested that poorly differentiated tumors have higher growth and recurrence rates than well-differentiated tumors do [17]. Previous study reported that down-regulation of MTSS1 expression might correlate with the transition of tumor cells from distinct epithelium-like morphology to less differentiated carcinomas [18]. Consistently, in our study, MTSS1 protein expression was lost more frequently in poorly differentiated tumors than in well-differentiated tumors, suggesting that MTSS1 is a differentiation marker for gastric cancer. However, another report reveals that an incremental increase in MTSS1 expression was detected from healthy normal liver donors to non tumor tissue specimens and then to their matched hepatocellular carcinoma tumor tissue specimens. Specifically, a higher level of MTSS1 expression was observed at the early stages of the disease, suggesting that MTSS1 may play an important role in promoting the early development of hepatocellular carcinoma [16]. Inasmuch as the role of MTSS1 has not been clearly defined to date because of contradicting published data, we speculate that the role of MTSS1 could be cancer or tissue type specific.

Risk factors for gastric cancers have been explored in a number of studies, including status of lymph nodes, depth of tumor invasion, age at diagnosis, TNM stage and some molecular markers [5,7,19-23]. In the present study, we confirmed that age, histology, nodal metastasis and TNM stage were independent predictors for gastric cancer. Furthermore, MTSS1 expression was found to be significantly correlated with prognosis in univariate survival analysis and it still kept its prognostic value in multivariate survival analysis. Positive associations between MTSS1 expression and other clinicopathologic features, such as tumor size, depth of tumor invasion, status of lymph nodes, TNM stage, differentiation and Lauren's classification were indicated. All these findings suggested that MTSS1 expression alone was a potential molecular marker for predicting outcome in patients who undergo gastrectomy for gastric cancer.

Metastasis is a fatal step in the progression of gastric cancer and has become one of the most challenging problems in tumor therapy [24]. The spread of tumor cells is a complicated and multistage process and requires altered expression of many different genes [25]. *Nixdorf et al *[13] indicated that down-regulation of MTSS1 occurred in BL17/2 bladder tumor cell lines with invasive abilities. Expression of MTSS1 has also been shown to be reduced in prostate cancer and can contribute to tumor growth and development, as well as metastasis [12]. In this study, we observed significantly lower expression of MTSS1 in the nodal metastases of gastric cancers than that in primary tumors. The high frequency of down regulation of MTSS1 expression in primary gastric tumors and lost MTSS1 expression in metastases and their direct association with poor outcome of gastric cancer indicated that MTSS1 could suppress tumor invasion and metastasis, and might be a candidate prognostic factor for lymph node metastasis and tumor progression. This notion is clearly supported by a recent study showing that biological overexpression of MTSS1 significantly suppressed the invasive, migratory, growth and adherence properties of a human breast cancer cell line [26]. However, *Bompard et al *[15] found that MTSS1 expression is not dependent on the metastatic state of the cells. We also observed MTSS1 expression in six nodal metastases but not in matched primary tumors. This discrepancy makes us presume that there is a possibility that the components which regulate MTSS1 may create specific interactions with different microenvironments between primary regions and target organs. Expression of MTSS1 is regulated by an epigenetic event that is differently represented in different sites. The mechanism for the down-regulation of MTSS1 to tumor progression, especially metastasis, is not clear. It was reported that its expression is regulated by DNA methylation, a potential hallmark of a gene likely to be involved in tumor initiation and progression [11]. Additionally, several studies indicated that MTSS1 may be due to actin binding, reorganisation or changes in cell adhesion or tyrosine phosphorylation upon loss of MTSS1 [27]. As reorganization of the actin cytoskeleton has been extensively studied in cancer and is the primary mechanism of cell motility and migration, which are critical steps, involved in tumor carcinogenesis [28]. Further functional investigations are worthwhile to explore the precise mechanism of the carcinogenic effect of MTSS1 with the goal of developing potential therapies targeting MTSS1 as an indicator for gastric cancer.

## Conclusion

To the best of our knowledge, this is the first report demonstrating the clinicopathological significance of MTSS1 expression in gastric cancer. Our results showed that loss of MTSS1 expression was associated with large tumor size, low differentiation, deep invasion level, advanced tumor stage, the presence of nodal metastasis, and poor outcome in patients who underwent gastrectomy. MTSS1 expression may serve as a useful biomarker for the prediction of outcome of gastric cancer.

## Competing interests

The authors declare that they have no competing interests.

## Authors' contributions

KL participated in the design of the study, carried out the mRNA expression of MTSS1 in GC, the immunohistochemistry of tissue microarrays and drafted the manuscript. GFW participated the immunohistochemistry analysis of MTSS1 in GC patients and assisted the analysis of data. HZD performed the Western Blotting test and assisted the collection of clinical data. YC participated evaluation of immunostaining. GZY and JJW participated in its design and coordination, and supervised the study. KL and GFW contributed equally to this work. All authors read and approved the final manuscript.

## Pre-publication history

The pre-publication history for this paper can be accessed here:

http://www.biomedcentral.com/1471-2407/10/428/prepub
